# Cancer prevention in Africa: liver cancer

**DOI:** 10.3332/ecancer.2019.950

**Published:** 2019-07-25

**Authors:** Paul Ndom

**Affiliations:** Yaoundé General Hospital, PO Box 5408, Yaounde, Cameroon

**Keywords:** liver cancer, prevention, Africa

## Abstract

Hepatocellular carcinoma is a common cancer in Africa. The risk factors are well known and avoidable in most cases (hepatitis B, hepatitis C, aflatoxin and alcohol). Vaccination against hepatitis B and the fight against aflatoxin are efficient contributions to the fight against liver cancer. The costly nature of these measures in Africa is an impediment to the fight against liver cancer in Africa.

## Background

Liver cancer stands as the seventh most common cancer in both genders worldwide and the second cause of deaths due to cancer. In 2012, the worldwide incidence of liver cancer was estimated at 782,451 new cases with a mortality of 745,533 cases [1]. In the same year, it was the fifth most common cancer in men with 554,369 cases, for a mortality of 521,041, while in women, it was the ninth most common cancer with 228,082 cases, for a mortality of 224,492 cases. These figures show that the number of deaths due to liver cancer almost equals the number of new cases [1].

In Africa, it is the fourth most common cancer in both genders, with 64,779 new cases for a mortality of 63,562 cases with a prevalence of about 56,736 cases in 2018. In men, it is the second most common cancer and the second cause of death due to cancer with 43,530 new cases (9.7%) (Table 1) [1].

Depending on the region, liver cancer is thought to result from the exposure to many risk factors. These factors vary from one place to another, depending on habits, lifestyle, food consumed and surrounding infections. Prevention of liver cancer requires knowledge of these risk factors and ways to avoid them.

## Risk factors and prevention

In Africa, the main risk factors for liver cancer are hepatitis B and C viral infections (HBV and HCV) (Table 2). Other risk factors include exposure to aflatoxin, alcohol induced liver cirrhosis and metabolic syndrome [2, 3]. They are summarised in Table 2.

## Chronic hepatitis B virus

Chronic hepatitis B virus (HBV) infection affects 350 million people worldwide and is the most common risk factor for hepatocellular Carcinoma (HCC) [6].

75 million people in Africa are affected by HBV. The main causes of high hepatitis B prevalence in Africa include inadequate information on the means of transmission, inaccurate estimates of the disease’s burden and insufficient vaccine coverage. HBV is transmitted through contact with the blood and body fluids of an infected person. However, the role of mother-to-child transmission is often underestimated. Unsafe injections from poorly sterilised needles and re-used equipment are a major source of new infections. According to the World Health Organization (WHO), 1.7 million people are infected via unsafe injection practices. These include scarification (for traditional protection and treatment) and cultural body-piercing and tattoo practices using razor blades and similar sharp objects that are contaminated with infected blood [7].

In November 2014, the WHO African Regional Committee endorsed a resolution for a hepatitis B control goal to reduce chronic HBV infection prevalence to less than 2% in children less than 5 years of age in all member States by 2020 [8].

Co-infection of HBV and HIV is not uncommon, affecting around 1% of people living with HBV infection (2.7 million people). Conversely, the global prevalence of HBV infection in HIV-infected persons is 7.4%. Since 2015, WHO has recommended treatment for everyone diagnosed with HIV infection, regardless of the stage of the disease. Tenofovir, which is among the first-line treatments against HIV infection, is also active against HBV [9].

Hepatitis B can be controlled through vaccination, treating infected individuals and interrupting the spread of the infection.

## Prevention of HBV

Prevention is aimed at eliminating or reducing the factors and conditions cited above. Vaccination against hepatitis B is the most effective method of preventing HBV infection in both new-borns and adults [7]. National hepatitis B vaccination program significantly reduces the prevalence and the incidence of HCC. However, more time is needed to reach the final results, because this program was first introduced across the world between 1982 and 1990 and most cases of HCC occur after the age of 40 years [10, 11].

## Hepatitis B vaccination

The backbone in the prevention of hepatitis B infection is vaccination. WHO recommends that all infants receive the hepatitis B vaccine as soon as possible after birth, preferably within 24 hours. In 2015, the global estimated prevalence of HBV infection in children under 5 years of age was about 1.3%, compared with about 4.7% in the pre-vaccination era. This low incidence can be attributed mainly to the widespread use of hepatitis B vaccine. The birth dose should be followed by two or three doses to complete the primary series. In most cases, one of the following two options is considered appropriate: a three-dose schedule of hepatitis B vaccine, with the first dose (monovalent) being given at birth and the second and third (monovalent or combined vaccine) given at the same time as the first and third doses of diphtheria, pertussis (whooping cough), and tetanus—(DTP) vaccine; or a four-dose schedule, where a monovalent birth dose is followed by three monovalent or combined vaccine doses, usually given with other routine infant vaccines. [8, 12, 13]

When the vaccine is taken properly and completely, it induces protective antibody levels in more than 95% of infants, children and young adults which last at least 20 years and probably lifelong. Thus, WHO does not recommend booster vaccination for persons who have completed the three-dose vaccination schedule. It is advised that children and adolescents younger than 18-year old living in countries with low or intermediate endemicity and who have not been previously vaccinated should receive the vaccine. In such settings, people in high risk groups should also be vaccinated as they are prone to infection. They include: people who frequently require blood or blood products, dialysis patients, recipients of solid organ transplantations; prison inmates; persons using intravenous drugs; household and sexual contacts of people with chronic HBV infection; people with multiple sexual partners; healthcare workers and others who may be exposed to blood and blood products through their work; travellers who have not completed their hepatitis B vaccination series, who should be offered the vaccine before leaving for endemic areas [12, 13].

Since 1982, over 1 billion doses of hepatitis B vaccine have been used worldwide with an excellent record of safety and effectiveness. Vaccination has reduced the rate of chronic infection to less than 1% among immunised children in many countries where between 8% 15% of children used to become chronically infected with the hepatitis B virus.

In 2015, global coverage with the third dose and the birth dose of hepatitis B vaccine reached 84% and 39%, respectively, with the WHO regions of the Americas and Western Pacific being the only regions that had wide coverage. Another effective method of HBV transmission prevention is the implementation of blood safety strategies, including quality-assured screening of all donated blood and blood components used for transfusion. Worldwide, in 2013, 97% of blood donations were screened and quality assured, but there continue to be lapses in the implementation. Other effective strategies are safe injection practices, eliminating unnecessary and unsafe injections. Globally, unsafe injections decreased from 39% in 2000 to 5% in 2010. Furthermore, safer sex practices such as minimising the number of partners and using barrier protective measures (condoms), also protect against transmission.

All 47 countries in the WHO Africa region have introduced hepatitis B into the routine infant immunisation schedule; 44 (94%) countries use pentavalent vaccine (diphtheria, tetanus, pertussis, *Haemophilus influenza* type B and hepatitis B vaccines) and 33 (70%) countries follow a three-dose schedule at 6, 10 and 14 weeks of age. As of December 2016, nine countries, representing 28% of the regional birth cohort, have introduced a universal hepatitis B vaccine birth dose (HepB-BD) policy. Two countries, Sao Tome and Principe and Mauritius, only provide HepB-BD for babies born to HBsAg-positive mothers (hepatitis B surface antigen) [13]. From the year 2000 to 2015, regional reported coverage with three doses of hepatitis B (HepB3) increased from 5% to 76%. However, since 2009, coverage has plateaued at 70%–75% which is below the global coverage of 2015 at 84%. The country-specific HepB3 coverage estimates for 2015 ranged from 16% in Equatorial Guinea to 98% in Rwanda, The Seychelles, Swaziland and United Republic of Tanzania; 16 (34%) countries reported national HepB3 coverage of at least 90%. Regional reported HepB-BD coverage increased from 0% in 2000 to 10% in 2015 although coverage has plateaued at 10% since 2010. This is below the 2015 global HepB-BD coverage of 39%. Among countries that have introduced the birth dose, HepB-BD coverage ranged from 19% in Angola to 99% in Algeria and Botswana). Algeria, Botswana, Cape Verde and The Gambia, all of which had introduced the birth dose over a decade ago, reported at least 90% national HepB-BD coverage. A recent situational report of the WHO African region indicated HepB-BD introduction has been recommended or is under consideration in Cameroon, Cote d’Ivoire, Guinea Bissau, Mozambique, Niger, the Republic of Congo, Sierra Leone, South Africa and Uganda. In Ethiopia and Gabon, HepB-BD introduction has been proposed for the next comprehensive multi-year plan. In Rwanda, the national Expanded Programme on Immunisation reported that it has received approval from the Ministry of Health but is waiting for endorsement from the Interagency Coordination Committee. Ghana has included HepB-BD introduction in its comprehensive multi-year strategic plan for immunization and the National Viral Hepatitis Control Plan, but so far, HepB-BD introduction has been postponed due to competing priorities. Countries have reported multiple barriers to HepB-BD introduction, including lack of financial support from Gavi, the vaccine alliance (10 countries), the need for evidence on the burden of chronic HBV infection and the risk of perinatal transmission in Africa (6 countries), insufficient cold chain storage (3 countries), lack of trained healthcare workers to attend births or conduct post-natal visits (2 countries) and a high proportion of home births (2 countries) [13].

More so, the price of screening and vaccination in resource limited countries is really a barrier to the involvement of the population in the fight against viral hepatitis.

## Perinatal transmission of HBV in the African region

Screening of pregnant women is the method of preventing mother-to-child transmission of hepatitis B. For women with positive hepatitis B surface antigen (HBsAg), HBeAg genotype and viral load tests are done. If the viral load is high (>200,000), the pregnant woman is placed on treatment from the third trimester of pregnancy to up to 12 weeks after delivery with nucleotide analogues (Lamivudine, Telbivudine and Tenofovir), which help to reduce failure associated with serovaccination. Also, at birth the newborn will receive a hepatitis B vaccine and anti HBs Immunoglobulin [14].

Four studies were identified that assessed perinatal transmission of HBV infection. In Burkina Faso, Cote d’Ivoire and Ghana, women with unknown or negative HIV status had a higher perinatal transmission rate of HBV when mothers expressed HBeAg or had a high HBV DNA viral load (≥10^4^ IU/ml). In Cote d’Ivoire, 9 (38%) of 24 infants born to HBsAg-positive/HBeAg-positive mothers tested HBsAg-positive at six weeks of age, compared with none (0%) of 142 infants born to HBsAg-positive/HBeAg-negative mothers. In Ghana, 5(5.2%) of 97 infants born to HBsAg-positive mothers tested HBV DNA positive at two weeks of age; the relative risk of perinatal transmission associated with high maternal HBV DNA viral load (≥10^4^ IU/ml) compared with low maternal HBV DNA viral load was 2.4 (95% CI:1.1–5.4, *p* = 0.048). One study from Burkina Faso reported that 7 (32%) of 22 infants born to HBsAg-positive/HBeAg-negative and 2 (29%) of 7 infants born to HBsAg-positive/HBeAg-positive mothers tested HBsAg-positive within 24 hours of birth [13].

## Antiviral treatment for HBV

Results from many studies suggest that antiviral therapy is very effective in controlling HBV infection and reducing the incidence of HCC. Patients receiving antiviral treatment, especially those in virologic suppression, less frequently develop HCC as opposed to untreated persons. However, the risk of developing HCC cannot be eliminated, implying that these have to be monitored throughout their lifetime until treatments are available to completely eradicate HBV from the liver.

## HCV infection

Patients with HCV infection have a 15- to 20-fold increase risk for developing HCC. Once HCV-related cirrhosis is established, HCC develops at an annual rate of 1%–8% (average, 3.5%).

The oncogenic risk for hepatitis C is lower than that of hepatitis B. Hepatitis B has a direct oncogenic effect on hepatocytes while some studies suggest that hepatitis C must cause liver cirrhosis before liver cancer [15].

## Prevention of HCV

HCV infection prevention is more challenging than that of HBV due to the absence of an effective vaccine, and requires a fundamental and comprehensive strategy, including: blood donation screening, safe injection and safe traditional excisions and scarification’s, tattooing and piercing [16].

## Antiviral treatment for HCV

The current treatments for HCV infection are direct acting antivirals. Combination therapy decreases the risk of HCC in patients with HCV-related cirrhosis, even without complete biochemical and virological clearing [17].

Identifying and monitoring all the cases of cirrhosis is an effective strategy in liver cancer prevention.

## Alcohol consumption

Ethanol is mainly metabolised in the liver, producing acetaldehyde and free radicals which cause liver injury and DNA damage through the increase of oxidative stress [18]. This risk is increased in many areas in Africa due to high consumption usually of cheaper and degraded /artificial /adulterated alcohols.

Measures such as price increment, increased taxation and custom duties on alcohol, and policies against artificial alcohols should be taken.

## Aflatoxin

Aflatoxin is secreted by a mushroom called Aspergillus flavus. This mushroom is found in several food items:

- Cereal grains: maize, rice
- Oleaginous grains: groundnut, sunflower
- Spices: Chili pepper, curry and ginger
- Tree nuts: almond, kernel and egusi
- Figs, dates, cocoa, coffee, cassava [19]

**Figure 1.**
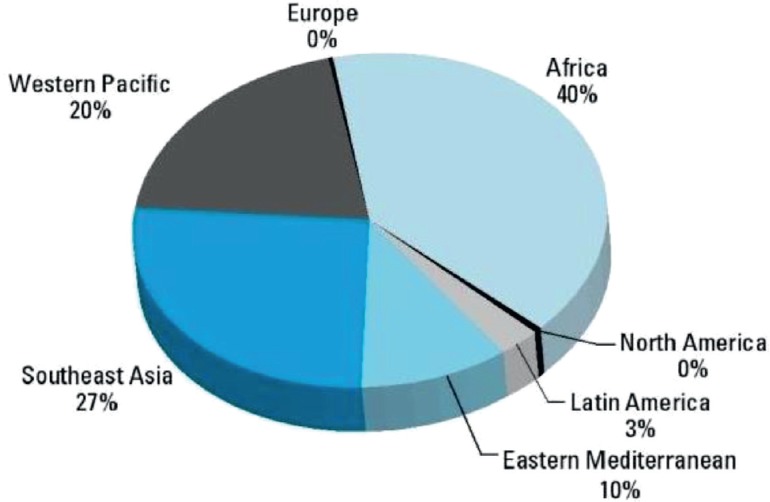
Distribution of HCC cases attributable to aflatoxin in different regions of the world [20].

Aflatoxin accounts for 30% of liver cancer. It causes a mutation in the p53 gene. Hot and humid climate favours its contamination. Figure 1 shows the distribution of HCC cases attributable to aflatoxin in different regions of the world.

**Figure 2.**
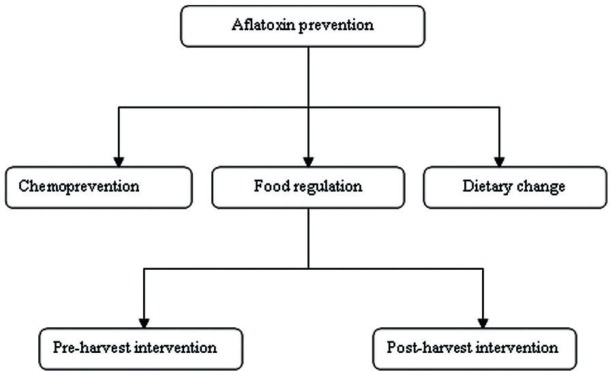
Aflatoxin prevention strategy [21].

Pre-harvest intervention consists in growing crops and post-harvest intervention in storage, processing, distribution and consumption (Figure 2).

## Fight against aflatoxin

Contamination usually occurs during cultivation and conservation of cereals, hence during planting, it is important to respect seasons, space plants with proper irrigation. Crop rotation and fallowing is also an efficient way to avoid this mushroom. Another means to fight aflatoxin is to consume dry grains and avoid parts with mould and when possible use electric or traditional driers. It is advised to consume well-cooked groundnuts and to place ready to consume groundnuts in a microwave before consumption as it is denatured by heat and also avoid conservation over long periods [22, 23].

## Conclusion and recommendations

Hepatocellular carcinoma is a common cancer in Africa. The risk factors are well known and avoidable in most cases (hepatitis B, hepatitis C, aflatoxin and alcohol). Vaccination against hepatitis B and the fight against aflatoxin are efficient contributions to the fight against liver cancer. The costly nature of these measures in Africa is an impediment to the fight against liver cancer in Africa.

Despite the introduction of hepatitis B by all countries in the region, for 31 countries (66%) HepB3 coverage is below the 90% recommended coverage level. Given the high chronic HBV infection prevalence throughout the region, particularly among pregnant women, and the importance of perinatal and early childhood transmission in intermediate and high endemicity settings, countries need to improve HepB3 coverage.

In some countries, high prevalence of home births, the lack of services available to reach infants born at home and unreliable vaccine supply have limited hepatitis B implementation.

We recommend a subvention for hepatitis B vaccination, to improve access to screening for hepatitis at a reduced cost, an effective fight against aflatoxin, a reduction in abusive alcohol consumption and education of the masses on risky behaviours.

## Conflicts of interest

There are no conflicts of interest with regard to the content of this article.

## Funding

This work was not supported by any funding.

## Figures and Tables

**Table 1.** Incidence and mortality of liver cancer in Africa [1].

	Incidence						Mortality					
	Both sexes		Males		Females		Both sexes		Males		Females	
	New cases	Cum. Risk 0–74 (%)	New cases	Cum. Risk 0–74 (%)	New cases	Cum. Risk 0–74 (%)	Deaths	Cum. Risk 0–74 (%)	Deaths	Cum. Risk 0–74 (%)	Deaths	Cum. Risk 0–74 (%)
Eastern Africa	11,550	0.52	7,011	0.66	4,539	0.40	11,251	0.52	6,799	0.67	4,452	0.40
Middle Africa	6,010	0.69	4,137	0.98	1,873	0.43	5,853	0.69	4,056	0.99	1,797	0.42
Northern Africa	27,935	1.71	19,912	2.50	8,023	0.96	27,505	1.69	19,570	2.47	7,935	0.95
Southern Africa	2,710	0.53	1,692	0.79	1,018	0.33	2,597	0.51	1,614	0.75	983	0.32
Western Africa	16,574	0.91	10,778	1.21	5,796	0.64	16,356	0.90	10,747	1.20	5,609	0.62

**Table 2.** Risk factors of liver cancer in Africa [4, 5].

Risk factor	Northern Africa (%)	Sub-Saharan Africa (%)
Hepatitis B	18	70
Hepatitis C	60	20
Alcohol	18	10
Aflatoxin	-	10
Metabolic syndrome, obesity and/or diabetes	18	<5
